# Increased risk of tinnitus following a trigeminal neuralgia diagnosis: a one-year follow-up study

**DOI:** 10.1186/s10194-020-01121-6

**Published:** 2020-05-06

**Authors:** Yen-Fu Cheng, Sudha Xirasagar, Tzong-Han Yang, Chuan-Song Wu, Yi-Wei Kao, Ben-Chang Shia, Herng-Ching Lin

**Affiliations:** 1 Department of Medical Research, Taipei Veterans General Hospital, Taipei, Taiwan; 2 Department of Otolaryngology-Head and Neck Surgery, Taipei Veterans General Hospital, Taipei, Taiwan; 3 Department of Speech, Language and Audiology, National Taipei University of Nursing and Health Sciences, Taipei, Taiwan; 4 Research Center of Sleep Medicine, College of Medicine, Taipei Medical University, Taipei, Taiwan; 5 Department of Health Services Policy and Management, Arnold School of Public Health, University of South Carolina, Columbia, USA; 6 Department of Otolaryngology, Taipei City Hospital, Taipei, Taiwan; 7 Big Data Research Center, Taipei Medical University, Taipei, Taiwan; 8 Graduate Institute of Business Administration, College of Management, Fu Jen Catholic University, New Taipei City, Taiwan; 9 Department of Health Care Administration, Taipei Medical University, 250 Wu-Hsing St, Taipei, 110 Taiwan; 10 Sleep Research Center, Taipei Medical University Hospital, Taipei, Taiwan

**Keywords:** Tinnitus, Trigeminal neuralgia, Trigeminal nerve

## Abstract

**Background:**

Tinnitus due to hyperactivity across neuronal ensembles along the auditory pathway is reported. We hypothesized that trigeminal neuralgia patients may subsequently suffer from tinnitus. Using nationwide, population-based data and a retrospective cohort study design, we investigated the risk of tinnitus within 1 year following trigeminal neuralgia.

**Methods:**

We used the Taiwan National Health Insurance Research Dataset, a claims database, to identify all patients diagnosed with trigeminal neuralgia from January 2001 to December 2014, 12,587 patients. From the remaining patients, we identified 12,587 comparison patients without trigeminal neuralgia by propensity score matching, using sex, age, monthly income, geographic region, residential urbanization level, and tinnitus-relevant comorbidities (hyperlipidemia, diabetes, coronary heart disease, hypertension, cervical spondylosis, temporomandibular joint disorders and injury to head and neck and index year). All study patients (*n* = 25,174) were tracked for a one-year period to identify those with a subsequent diagnosis of tinnitus over 1-year follow-up.

**Results:**

Among total 25,174 sample patients, the incidence of tinnitus was 18.21 per 100 person-years (95% CI = 17.66 ~ 18.77), the rate being 23.57 (95% CI = 22.68 ~ 24.49) among patients with trigeminal neuralgia and 13.17 (95% CI = 12.53 ~ 13.84) among comparison patients. Furthermore, the adjusted Cox proportional hazard ratio for tinnitus in the trigeminal neuralgia group was 1.68 (95% CI = 1.58 ~ 1.80) relative to the comparison cohort.

**Conclusions:**

We found a significantly increased risk of tinnitus within 1 year of trigeminal neuralgia diagnosis compared to those without the diagnosis. Further studies in other countries and ethnicities are needed to explore the relationship between trigeminal neuralgia and subsequent tinnitus.

## Introduction

Tinnitus is the auditory phantom sensation of sound in the absence of external stimuli. About 10% of the population suffers from tinnitus, making it one of the most common health conditions in the world [1, 2]. The most common cause of tinnitus is tinnitus associated with hearing loss caused by noise overexposure and aging. However, tinnitus can result from non-otologic causes, such as head and neck trauma [3], temporomandibular disorders [4–7], and cervical spine disorders [8, 9]. A certain percentage of patients find their tinnitus provoked by movement of or applying pressure to the head and neck region [10, 11]. Research has shown that the somatic origins of tinnitus may be due to interactions between somatic and auditory neuronal pathways in the central nervous system, indicating the role of somatosensory components in some cases of tinnitus [10, 12–16].

Trigeminal neuralgia is a common cause of chronic orofacial pain due to inflammation or other pathology of the trigeminal nerve. The trigeminal nerve, which divides into three branches, the ophthalmic (V1), maxillary (V2), and mandibular (V3) branches, is responsible for the sensory supply of the orofacial region. Trigeminal neuralgia is characterized by extremely disturbing, sporadic, and recurrent episodes of burning facial pain. While it is typically characterized by paroxysmal attacks of facial pain, atypical trigeminal neuralgia may manifest as a less intense condition associated with a constant background pain without intervals of relief.

Trigeminal neuralgia, like other pain disorders of the head and neck region such as temporomandibular disorders (TMD) and cervical spine disorders (CSD), is thought to be associated with transmission of nociceptive inputs through the trigeminal nerve which converge with other somatosensory pathways in the brainstem [17–19]. There is increasing evidence of tinnitus originating from hyperactivity across neuronal ensembles along the auditory pathway [20–22], and from functional and anatomical interchanges between the somatosensory and auditory pathways in the brainstem [14, 23–25]. Previous animal studies have demonstrated that the trigeminal nerve input interacts with the neural activity of the central auditory pathways related to sound perception at the level of dorsal cochlear nucleus [13, 26]. This may explain the mechanism of tinnitus experienced by some patients with trigeminal nerve pathology. Pathophysiologic involvement of the trigeminal system in tinnitus is also supported by clinical observations that tinnitus sensation can be elicited or is reported to be triggered by certain face and jaw movements in some patients [10, 11, 27].

Therefore, it is reasonable to hypothesize a prospective or comorbid association between trigeminal neuralgia and tinnitus. To the best of our knowledge, there is no documented study that explored tinnitus risk following a diagnosis of trigeminal neuralgia. This nationwide, population-based, retrospective cohort study was carried out to investigate the risk of tinnitus within 1 year following a diagnosis of trigeminal neuralgia.

## Methods

### Database

Data for this study was accessed from the Taiwan National Health Insurance Research Dataset (NHIRD). The NHIRD is a claims database extracted from Taiwan’s National Health Insurance (NHI) system claims data which has all medical claims of about 23.95 million NHI enrollees in 2018 (99% of the Taiwanese population). The NHIRD allows researchers in Taiwan access to de-identified longitudinal claims data on all medical care utilized by 1 million randomly selected enrollees, all care utilization since the initiation of NHI in 1995. The data present an opportunity to explore the relationship between trigeminal neuralgia and subsequent occurrence of tinnitus. The study was approved by the Institutional Review Board of Taipei Medical University (TMU-JIRB N201712044).

### Study sample

The study was designed as a retrospective cohort study. To select study patients, we first identified 132,803 patients who received their first diagnosis of trigeminal neuralgia (ICD-9-CM code 350.1) at outpatient facilities (doctor’s offices or outpatient departments of hospitals) between 1 January 2001 and 31 December 2014. We assigned the date of their first-time diagnosis of trigeminal neuralgia as their index date. We excluded 1859 patients less than 18 years of age, and 118,357 patients who had received a diagnosis of tinnitus (ICD-9-CM code 388.3) within the year prior to the index date. Ultimately, the selected trigeminal neuralgia (study) cohort included 12,587 patients.

We identified comparison patients from the Registry of beneficiaries included in the NHIRD. After excluding patients with trigeminal neuralgia and those aged less than 18 years of age, we used propensity-score matching to select 12,587 comparison patients who matched the study patients on sex, age group, monthly income, geographic region, urbanization level of the patient’s residence (5 levels, 1 = most urbanized, 5 = least urbanized), vertigo-relevant co-morbidities (hyperlipidemia, diabetes, coronary heart disease, hypertension, cervical spondylosis, temporomandibular joint disorders and injury to head and neck), and index year. The index year for patients of the study cohort was the year when they first received the diagnosis of trigeminal neuralgia, and the date was their index date. For comparison cohort patients, their index year was the year of their matched trigeminal neuralgia case after ensuring they had at least one episode of medical care utilization in the index year. We assigned their index date as the date of first utilization of medical care in the index year. We further screened the comparison patients to exclude those who had ever received a diagnosis of tinnitus in the year prior to their index date.

### Outcome variable

The outcome variable of interest was tinnitus occurring during one-year follow-up from the index date. Patient (*n* = 25,174) were tracked for a one-year period following their index date to identify any claim with a diagnosis of tinnitus.

### Statistical analysis

We used the SAS statistical package (SAS System for Windows, Version 8.2, Cary NC, USA) for statistical analyses. We used the Kaplan-Meier curves with log-rank test to examine the differences in one-year, tinnitus-free survival between the study cohort and comparison cohort. Cox proportional hazards regression analysis was used to estimate the one-year adjusted risk of tinnitus following trigeminal neuralgia. We verified that the proportional hazards assumption was satisfied based on survival curves for both strata (study and comparison cohorts) showing hazard functions that were proportional over time. Adjusted hazard ratio (HR) along with 95% confidence intervals (CI) was used to estimate the risk of tinnitus, with a two-sided *p* value < 0.05 considered as statistically significant.

## Results

Table 1 presents the sample distribution, categorized by trigeminal neuralgia status on sociodemographic characteristics and co-morbidities. Of the total sampled patients, the mean age was 54.6 years old (standard deviation = 14.7 years old); 54.5 and 54.7 for study patients and comparison patients, respectively (*p* = 0.237). After propensity-score matching, we found statistically significant differences in sex (*p* = 0.016), monthly income (*p* = 0.011) hypertension (*p* = 0.008) and hyperlipidemia (*p* < 0.001), cervical spondylosis (*p* < 0.001), temporomandibular joint disorders (*p* < 0.001) and injury to head and neck (*p* < 0.001) between study and comparison patients. The two groups were similar on age, geographic region, diabetes and CHD.

**Table 1:** Demographic characteristics of patients with trigeminal neuralgia and comparison patients in Taiwan, 2001–2014 (total patients = 25,174)

Variable	Patients with trigeminal neuralgia (*n* = 12,587)		Comparison patients (*n =* 12,587)		*P* value
	Total No.	%	Total No.	%	
Males	4495	35.7	4680	37.2	0.016
Age, Mean (SD)	54.5 (14.8)		54.7 (14.7)		0.237
Hypertension	4910	39.0	4703	37.4	0.008
Coronary heart disease	2261	18.0	2199	17.5	0.314
Hyperlipidemia	3405	27.1	3655	29.0	< 0.001
Diabetes	2160	17.2	2153	17.1	0.920
Cervical spondylosis	1611	12.8	712	5.7	< 0.001
Injury to head and neck	118	0.9	59	0.5	< 0.001
Temporomandibular joint disorders	1126	8.9	161	1.3	< 0.001

*D* standard deviation

**Fig. 1:**
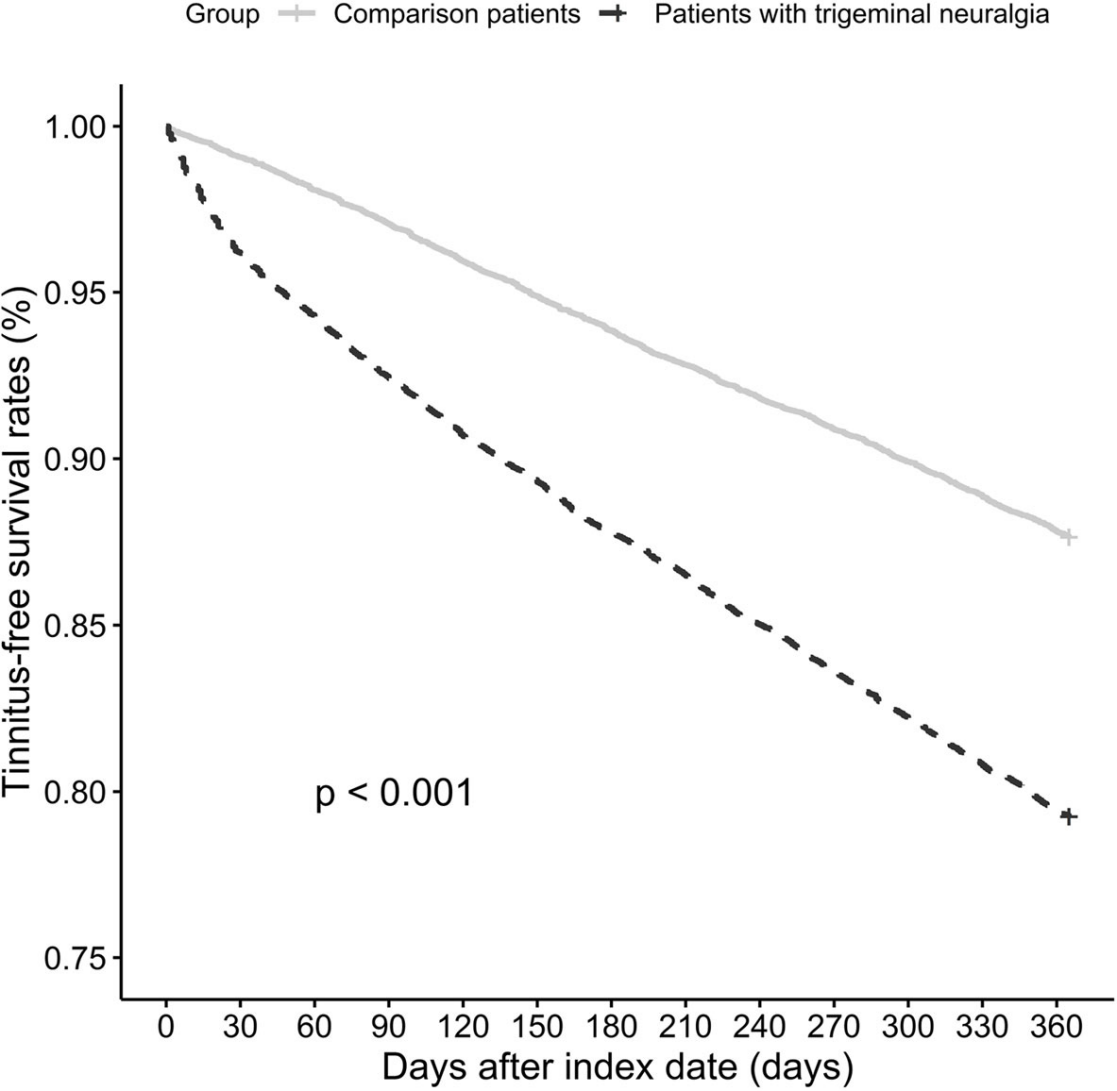
Tinnitus-free survival rates for patients with trigeminal neuralgia and comparison patients (x axis = days after index date, y axis = tinnitus-free survival rate)

Table 2 shows the overall incidence of tinnitus during one-year follow-up, and incidence among study patients and comparison patients. Among the total 25,174 sample patients, incidence of tinnitus was 18.21 per 100 person-years (95% CI = 17.66 ~ 18.77), 23.57 (95% CI = 22.68 ~ 24.49) among the trigeminal neuralgia group, and 13.17 (95% CI = 12.53 ~ 13.84) among the comparison group. The Kaplan-Meier log-rank test suggested that patients with trigeminal neuralgia have a significantly lower, 1-year tinnitus-free survival compared to the comparison group (*p* < 0.001). Figure 1 presents the Kaplan-Meier survival curves showing tinnitus-free survival among the two groups.

**Table 2:** Incidence rate, crude and adjusted hazard ratios for tinnitus among the sampled patients

Tinnitus occurrence over 1-year follow-up	Total *(n =* 25,174)		Patients with trigeminal neuralgia (*n* = 12,587)		Comparison groups (*n =* 12,587)	
	No.	%	No.	%	No.	%
Yes	4168	16.6	2612	20.8	1556	12.4
Incidence per 100 person-years (95%CI)	18.2 (17.7 ~ 18.8)		23.6 (22.7 ~ 24.5)		13.2 (12.5 ~ 13.8)	
Crude HR (95% CI)	–		1.78 (1.67, 1.90)***		1.00	
Adjusted ^a^ HR (95% CI)	–		1.68 (1.58, 1.80)***		1.00	

*Notes:* *** indicates *p* < 0.001. HZ = hazard ratio

^a^Adjustment for patient’s sex, age, urbanization level, monthly income and geographic region, and hypertension, hyperlipidemia, coronary heart disease, diabetes, cervical spondylosis, temporomandibular joint disorders and injury to head and neck

Table 2 also shows that the unadjusted HR for tinnitus of the trigeminal neuralgia group relative to the comparison group was 1.78 (95% CI = 1.67 ~ 1.90) during 1-year follow-up. The adjusted HR was 1.68 (95% CI = 1.58 ~ 1.80) after adjusting for age, sex, monthly income, geographic region, hyperlipidemia, diabetes, coronary heart disease, hypertension, cervical spondylosis, temporomandibular joint disorders and injury to head and neck.

Table 3 shows the relationship between trigeminal neuralgia and tinnitus stratified by surgical intervention. We found that the unadjusted HR for tinnitus for patients with trigeminal neuralgia who underwent surgical interventions relative to those without intervention was 2.91 (95% CI = 1.31 ~ 6.49). However, statistical significance of the association was not sustained after adjusting for age, sex, monthly income, geographic region, hyperlipidemia, diabetes, coronary heart disease, hypertension, cervical spondylosis, temporomandibular joint disorders and injury to head and neck (adjusted HR = 2.20, 95% CI = 0.98 ~ 4.91). It is possible however, that loss of statistical significance with adjustment for covariates may be due to the inadequate statistical power.

**Table 3:** Incidence rate, crude and adjusted hazard ratios for tinnitus among the sampled patients stratified by surgical intervention

Tinnitus occurrence over 1-year follow-up	Patients with trigeminal neuralgia who underwent surgical interventions (*n* = 19)		Patients with trigeminal neuralgia who did not undergo surgical interventions (*n* = 12,568)		Comparison groups (*n =* 12,587)	
	No.	%	No.	%	No.	%
Yes	6	31.6	2606	20.7	1556	12.4
Incidence per 100 person-years (95%CI)	38.5 (15.6 ~ 80.1)		23.6 (22.7 ~ 24.5)		13.2 (12.5 ~ 13.8)	
Crude HR (95% CI)	2.91 (1.31, 6.49)**		1.78 (1.67, 1.9)***		1.00	
Adjusted ^a^ HR (95% CI)	2.20 (0.98, 4.91)		1.68 (1.58, 1.8)***		1.00	

*Notes:* ** indicates *p* < 0.01; *** indicates *p* < 0.001; *HZ* hazard ratio

^a^Adjustment for patient’s sex, age, urbanization level, monthly income and geographic region, and hypertension, hyperlipidemia, coronary heart disease, diabetes, cervical spondylosis, temporomandibular joint disorders and injury to head and neck

Table 4 presents the incidence and HR of tinnitus between study and comparison patients by age group. It indicates that there was a consistent significant relationship between trigeminal neuralgia and tinnitus across all age groups. We found that the relationship to be the most pronounced among those < 45 years, with an adjusted HR of 1.91 (95% CI: 1.65 ~ 2.22) for patients with trigeminal neuralgia relative to the comparison group.

**Table 4:** Incidence rate, crude and adjusted hazard ratios for tinnitus among the sampled patients according to age group

Tinnitus occurrence over 1-year follow-up	< 45		45 ~ 64		> 64	
	Patients with trigeminal neuralgia (*n* = 2933)	Comparison groups (*n =* 2862)	Patients with trigeminal neuralgia (*n* = 6195)	Comparison groups (*n =* 6196)	Patients with trigeminal neuralgia (*n* = 3459)	Comparison groups (*n =* 3529)
	*n*, %		*n*, %		*n*, %	
Yes	544 (18.5)	270 (9.4)	1261 (20.4)	711 (11.5)	807 (23.3)	575 (16.3)
Incidence per 100 person-years (95%CI)	20.9 (19.2 ~ 22.7)	9.9 (8.8 ~ 11.1)	23.1 (21.8 ~ 24.3)	12.2 (11.3 ~ 13.1)	26.9 (25.1 ~ 28.8)	17.8 (16.3 ~ 19.3)
Crude HR (95% CI)	2.09 (1.81, 2.42)***	1.00	1.89 (1.72, 2.07)***	1.00	1.51 (1.36, 1.68)***	1.00
Adjusted ^a^ HR (95% CI)	1.91 (1.65, 2.22)***	1.00	1.78 (1.62, 1.95)***	1.00	1.45 (1.30, 1.62)***	1.00

*Notes:* *** indicates *p* < 0.001. *HZ* hazard ratio

^a^Adjustment for patient’s sex, age, urbanization level, monthly income and geographic region, and hypertension, hyperlipidemia, coronary heart disease, diabetes, cervical spondylosis, temporomandibular joint disorders and injury to head and neck

Table 4 presents the incidence and HR of tinnitus between the study and comparison patients classified by age group. The results show a consistent and significant relationship between trigeminal neuralgia and tinnitus within all age groups. The association showed highest effect size among the age group of < 45 years, with an adjusted HR of 1.91 (95% CI: 1.65 ~ 2.22) compared to a HR of 1.78 for the age groups of 45 ~ 64 and 1.45 for the > 65 age group.

## Discussion

To our knowledge, this is the first population-based study to investigate the relationship between trigeminal neuralgia and subsequent appearance of tinnitus. Based on longitudinal data available in the National Health Insurance Research Database (NHIRD) of Taiwan, we find that trigeminal neuralgia is significantly associated with tinnitus in the year following the diagnosis.

While tinnitus has been traditionally associated with otologic conditions such as noise-induced hearing loss, a growing body of evidence has shown that convergence of auditory and somatosensory pathways in the brain stem also plays an important role in the pathogenesis of tinnitus [26–29]. The auditory system processes sound signals arising in the ear and transmits them to the cortex. The cochlear nucleus is the central site for multisensory integration of inputs originating in sources other than the auditory nerve, namely, somatosensory inputs from the trigeminal ganglia, cervical dorsal root ganglia, spiral trigeminal nucleus, and dorsal column nuclei [15, 23–25, 30]. Therefore, pathological changes in the peripheral somatosensory structures in the head and neck region may influence the auditory processing of inputs at the level of the cochlear nucleus. Trigeminal neuralgia and other tinnitus-causing somatosensory disorders such as TMD or CSD share some neurophysiological characteristics in transmitting the nociceptive inputs in the central nervous system. Therefore, disruptions in trigeminal inputs that are relayed in the cochlear nucleus may significantly influence the higher-order neuronal output, causing changes to the output transmitted on to the higher auditory pathways, causing phantom sound that is experienced as tinnitus.

Microvascular constriction within the inner ear has been proposed as a possible mechanism for tinnitus caused by disruption of sensorineural input from the auditory pathway [31–37]. Microvascular constriction affecting the inner ear may also affect the trigeminal nerve in its anatomical course through the inner ear, causing both trigeminal neuralgia and tinnitus as part of the same pathophysiology. Potentially this could underlie the observed comorbid occurrence of tinnitus and trigeminal neuralgia in some patients [31–37]. Disruptions of the trigeminal nerve caused by neuralgia may also induce or contribute to tinnitus by affecting the vasculature of the inner ear. The trigeminal nerve is the source of innervation for blood vessels around the spiral modiolus and the stria vascularis of inner ear [31, 38]. The major function of the stria vascularis is to regulate the ionic composition of the endolymph, to maintain high potassium concentrations to sustain the endolymphatic potential necessary for sound transduction. Disruption of the stria vascularis causes reduced perception of sounds and altered auditory processing, potentially leading to tinnitus [32].

A strength of this study is that more than 99% of Taiwan’s population is enrolled in the NHI, and the health system, both provider availability and health insurance features are designed to be maximally affordable and accessible to all citizens regardless of socio-economic status. Therefore, apart from being representative of the entire Taiwanese population, the study has minimal potential for bias due to differential healthcare utilization rates among the study and control groups. The advantage of a population-based study is the ability to achieve a large sample size with minimal selection bias and participation bias.

Despite its strengths, a cautionary note due to some limitations is warranted. First, epidemiological association does not prove biological causation. Biomedical studies are needed to explain the causal mechanisms underlying the observed association. Second, under-ascertainment of tinnitus is a potential limitation because only subjects who sought medical care can be identified from a claims database. Such bias would lead to underestimation of the association between trigeminal neuralgia and tinnitus. Finally, the dataset used in this study does not provide information on types of TN, clinical response to the typical drugs used for TN, treatment strategy, cranial autonomic features and neurovascular compression which could provide some insight into the pathophysiological context and etiology of the observed association. Additionally, the absence of data on other potential risk factors and comorbidities, such as lifestyle, tobacco or other psychoactive substance use, anxiety, depression, attention disorders and sleep disorders also limits the strength of study findings.

## Conclusion

Our findings suggest that trigeminal neuralgia may be associated with subsequent development of tinnitus in the following year. Further studies in other regions and ethnicities are needed to confirm the observed association. Biomedical studies are needed to investigate the causal pathways involved in the development of tinnitus among trigeminal neuralgia patients.

## Data Availability

The National Health Insurance Research Database, which has been transferred to the Health and Welfare Data Science Center (HWDC). Interested researchers can obtain the data through formal application to the HWDC, Department of Statistics, Ministry of Health and Welfare, Taiwan (http://dep.mohw.gov.tw/DOS/np-2497-113.html).
