# Correction to: Increased risk of tinnitus following a trigeminal neuralgia diagnosis: a one-year follow-up study

**DOI:** 10.1186/s10194-020-01184-5

**Published:** 2020-09-18

**Authors:** Yen-Fu Cheng, Sudha Xirasagar, Tzong-Han Yang, Chuan-Song Wu, Yi-Wei Kao, Ben-Chang Shia, Herng-Ching Lin

**Affiliations:** 1 Department of Medical Research, Taipei Veterans General Hospital, Taipei, Taiwan; 2 Department of Otolaryngology-Head and Neck Surgery, Taipei Veterans General Hospital, Taipei, Taiwan; 3 Department of Speech, Language and Audiology, National Taipei University of Nursing and Health Sciences, Taipei, Taiwan; 4 Research Center of Sleep Medicine, College of Medicine, Taipei Medical University, Taipei, Taiwan; 5 Faculty of Medicine, School of Medicine, National Yang-Ming University, Taipei, Taiwan; 6 Institute of Brain Science, National Yang-Ming University, Taipei, Taiwan; 7 Department of Health Services Policy and Management, Arnold School of Public Health, University of South Carolina, Columbia, USA; 8 Department of Otolaryngology, Taipei City Hospital, Taipei, Taiwan; 9 Big Data Research Center, Taipei Medical University, Taipei, Taiwan; 10 Graduate Institute of Business Administration, College of Management, Fu Jen Catholic University, New Taipei City, Taiwan; 11 Department of Health Care Administration, Taipei Medical University, 250 Wu-Hsing St, Taipei, 110 Taiwan; 12 Sleep Research Center, Taipei Medical University Hospital, Taipei, Taiwan

**Correction to: J Headache Pain 21, 46 (2020)**

**https://doi.org/10.1186/s10194-020-01121-6**

Following publication of the original article [1], we were notified that the following affiliations need to be added for the first author Yen-Fu Cheng:

- Faculty of Medicine, School of Medicine, National Yang-Ming University, Taipei, Taiwan
- Institute of Brain Science, National Yang-Ming University, Taipei, Taiwan

## References

[1] Cheng Y (2020). Increased risk of tinnitus following a trigeminal neuralgia diagnosis: a one-year follow-up study. J Headache Pain.

